# Defining a serum cortisol cutoff level post-CRH stimulation for diagnosing ACTH deficiency: A retrospective study validated by a nationwide registry

**DOI:** 10.3389/fendo.2026.1741709

**Published:** 2026-02-02

**Authors:** Tetsushi Izuchi, Shintaro Iwama, Ayana Yamagami, Tomoko Kobayashi, Koji Suzuki, Takanori Murase, Masahiko Ando, Shingo Murasawa, Kosuke Mukai, Hiroshi Takagi, Hidenori Fukuoka, Tomoko Handa, Takeshi Onoue, Takashi Miyata, Mariko Sugiyama, Daisuke Hagiwara, Hidetaka Suga, Ryoichi Banno, Shigeyuki Tahara, Mitsuru Nishiyama, Kazunori Kageyama, Michio Otsuki, Hiroshi Arima

**Affiliations:** 1 Department of Endocrinology and Diabetes, Nagoya University Graduate School of Medicine, Nagoya, Japan; 2 Center for Advanced Medicine and Clinical Research, Nagoya University Hospital, Nagoya, Japan; 3 Department of Endocrinology and Metabolism, Hirosaki University Graduate School of Medicine, Hirosaki, Japan; 4 Department of Metabolic Medicine, the University of Osaka Graduate School of Medicine, Osaka, Japan; 5 Department of Endocrinology and Diabetology, Nagoya City University East Medical Center, Nagoya, Japan; 6 Division of Diabetes and Endocrinology, Kobe University Hospital, Kobe, Japan; 7 Department of Clinical Research Education, Nagoya University Graduate School of Medicine, Nagoya, Japan; 8 Research Center of Health, Physical Fitness and Sports, Nagoya University, Nagoya, Japan; 9 Department of Neurological Surgery, Nippon Medical School Musashikosugi Hospital, Kawasaki, Japan; 10 Department of Endocrinology, Metabolism and Nephrology, Kochi Medical School, Kochi, Japan; 11 Division of Diabetes, Metabolism, and Endocrinology, Faculty of Medicine, Tohoku Medical and Pharmaceutical University, Sendai, Japan; 12 Department of Endocrinology, Tokyo Women’s Medical University, Tokyo, Japan

**Keywords:** Adenohypophysis, corticorelin, corticotropin-releasing hormone, hydrocortisone, hypopituitarism

## Abstract

**Background:**

The corticotropin-releasing hormone (CRH) stimulation test is used to diagnose adrenocorticotropic hormone (ACTH) deficiency; however, the serum cortisol cutoff value indicating impaired response on this test (18 µg/dL [approximately 500 nmol/L]) was established from the insulin tolerance test. We aimed to define a serum cortisol cutoff after CRH stimulation to diagnose ACTH deficiency.

**Methods:**

Patients who underwent CRH stimulation at Nagoya University Hospital from 2016 to 2022 were divided retrospectively into two groups based on the need for hydrocortisone replacement at final follow-up (discovery cohort). Plasma ACTH and serum cortisol levels were measured at baseline and 30, 60, 90, and 120 minutes post-CRH administration using a current monoclonal antibody-based cortisol assay. The optimal cortisol cutoffs at each time point were determined by receiver operating characteristic (ROC) analysis. These cutoffs were validated using a nationwide disease registry in Japan (validation cohort).

**Results:**

In the discovery cohort (n = 227), cortisol levels were significantly higher in patients who did not receive hydrocortisone therapy (n = 136) than in those who did (n = 91) at all time points (p < 0.001). ROC analysis revealed that a 30-minute post-CRH cortisol level of 12.6 µg/dL (347.6 nmol/L) provided the best diagnostic performance to identify patients not requiring hydrocortisone therapy (sensitivity: 88.2%; specificity: 92.3%; AUC: 0.969). In the validation cohort (n = 52), this cutoff was confirmed as optimal (sensitivity: 81.0%; specificity: 86.4%; accuracy: 83.7%).

**Conclusion:**

A 30-minute post-CRH serum cortisol level of 12.6 µg/dL is a useful cutoff for diagnosing ACTH deficiency.

## Introduction

Adrenocorticotropic hormone (ACTH) deficiency is a potentially life-threatening condition that can lead to adrenal crisis if not managed appropriately. Therefore, accurate assessment of the hypothalamic–pituitary–adrenal (HPA) axis is crucial. The insulin tolerance test (ITT) is considered a reliable method for evaluating HPA axis function (1–6); however, due to induced hypoglycemia, it carries significant risks and has limited clinical applicability. In particular, the ITT is contraindicated in patients with a history of ischemic heart diseases or in the elderly (1, 4, 6).

In practice, a serum cortisol cutoff level of 18 µg/dL (~500 nmol/L) is commonly used to diagnose adrenal insufficiency based on responses to the ITT or ACTH stimulation (cosyntropin test) (3, 5–8). This cutoff originates from an earlier study in which patients with hypopituitarism (n = 22) showed peak cortisol levels ranging from < 1.0 to 9.0 µg/dL (< 27.6 to 248.3 nmol/L) on the ITT, whereas healthy controls (n = 44) had levels of 18.0–30.0 µg/dL (496.6 and 827.6 nmol/L) (9). However, since 2016, serum cortisol assays have shifted from polyclonal antibody-based immunoassays to newer methods utilizing monoclonal antibodies (10–12). These newer assays yield serum cortisol concentrations approximately 20% lower than those obtained by conventional methods (11, 13, 14).

The corticotropin-releasing hormone (CRH) stimulation test, an alternative method for assessing ACTH secretion, is safer and better tolerated than the ITT, particularly in elderly individuals (15–18). While a previous study reported a cortisol cutoff level of 17.5 µg/dL (482.8 nmol/L) for diagnosing central adrenal insufficiency with the CRH stimulation test, the data were based on polyclonal antibody-based immunoassays (18). Thus, no standardized serum cortisol cutoff based on current monoclonal antibody-based immunoassays has been established for diagnosing ACTH deficiency with the CRH stimulation test.

The aim of this study was to determine the serum cortisol cutoff after CRH stimulation that identifies patients with central adrenal insufficiency requiring hydrocortisone (HC) replacement therapy, based on retrospective data obtained at our institution since the implementation of a monoclonal antibody-based cortisol assay. In addition, we validated the utility of this cutoff using data from a nationwide registry for hypothalamic–pituitary disorders, established in 2021 by the Research Group on Hypothalamic–Pituitary Dysfunctions of the Ministry of Health, Labour and Welfare, Japan.

## Materials and methods

### Study design

This study consisted of two parts. First, CRH stimulation test data collected at Nagoya University Hospital (discovery cohort) were analyzed to determine a serum cortisol cutoff level for identifying patients who require HC replacement therapy. Second, CRH stimulation test data from a nationwide registry on hypothalamic–pituitary disorders (validation cohort) were analyzed to verify the validity of the cutoff level determined in the discovery cohort.

### Patients in the discovery cohort

**Figure 1:**
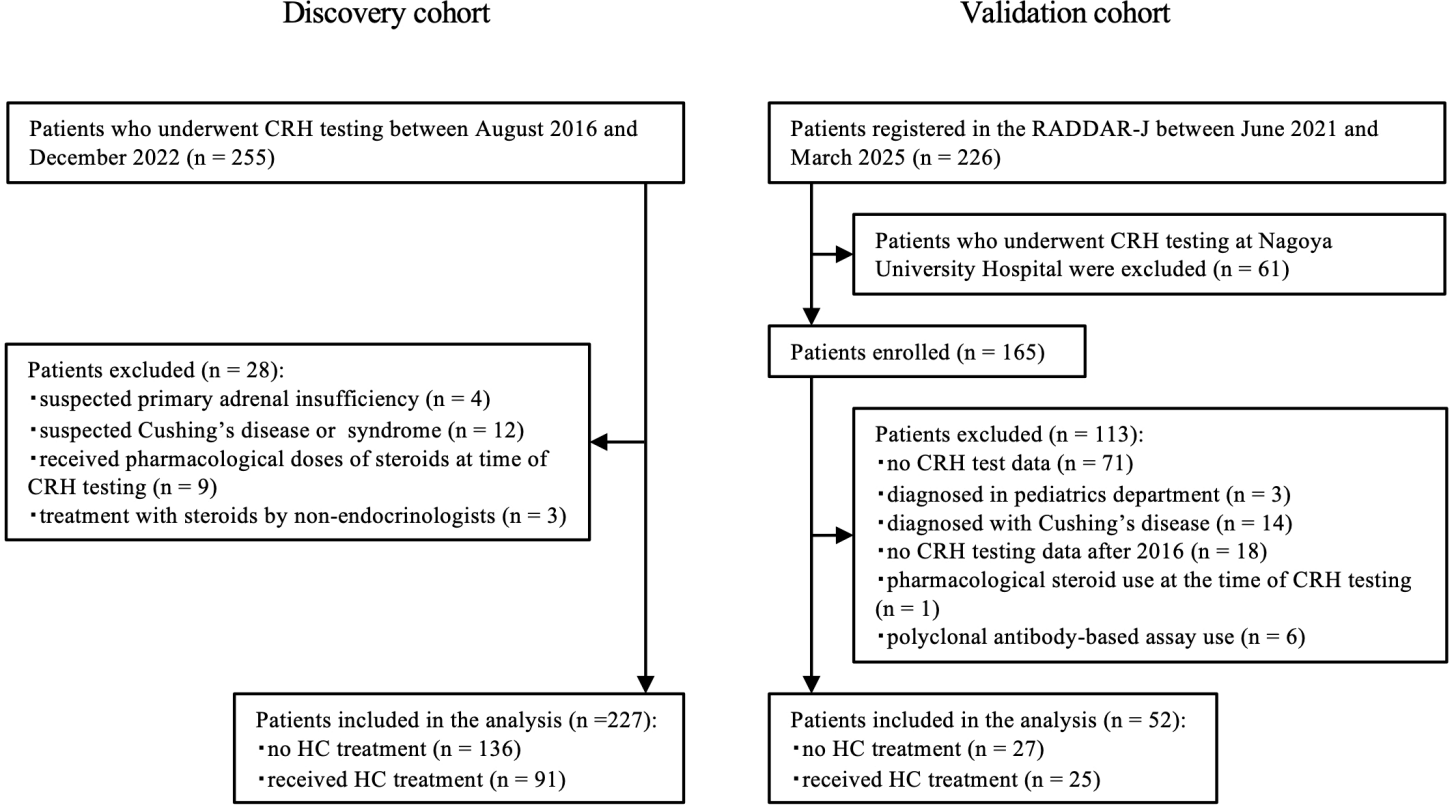
Flow diagram of patient enrollment in the discovery cohort and the validation cohort. Flow diagram of patient enrollment in the discovery and validation cohort. CRH, corticotropin-releasing hormone; HC, hydrocortisone; RADDAR-J, Rare Disease Data Registry of Japan.

Patients who underwent the CRH stimulation test at the Department of Endocrinology and Diabetes, Nagoya University Hospital between August 1, 2016 and December 31, 2022 were included in the discovery cohort (**Figure 1**). To examine the cutoff value of the CRH stimulation test in individuals with ACTH deficiency, we first excluded cases suspected of having primary adrenal insufficiency based on clinical information, including elevated ACTH and low cortisol levels. The other exclusion criteria were as follows: suspected Cushing’s disease or syndrome, pharmacological steroid use at the time of the CRH stimulation test, and initiation of HC therapy as advised by non-endocrinologists. The follow-up period was defined from the date of the CRH stimulation test to July 1, 2024 or the last clinical visit. Patients were classified into two groups based on the need for HC replacement therapy at the final follow-up: those not requiring HC [HC (–) group] and those requiring HC therapy [HC (+) group]. Both daily and intermittent (as needed) HC therapy users were included in the HC (+) group. For patients who underwent surgery or radiotherapy involving the pituitary gland or hypothalamus, the follow-up period ended prior to the intervention. For those who received steroid therapy for other diseases, the follow-up period ended on the date of steroid initiation. The need for HC treatment was comprehensively assessed by attending physicians affiliated with the Department of Endocrinology and Diabetes, based on CRH stimulation test results, clinical symptoms observed during the follow-up period, and relevant laboratory findings.

### Patients in the validation cohort

Patients registered in the nationwide Rare Disease Data Registry of Japan (RADDAR-J) established by the Research Group on Hypothalamic–Pituitary Dysfunctions of the Ministry of Health, Labour and Welfare, Japan in June 2021 were included in the validation cohort (**Figure 1**). Patients who underwent the CRH stimulation test between 2016 (when the new cortisol measurement assays were launched) and March 2025 were included to verify the diagnostic accuracy of the cutoff values obtained from the discovery cohort. The exclusion criteria were as follows: CRH stimulation test administered at Nagoya University Hospital (to avoid overlap with the discovery cohort), missing CRH stimulation test data, treatment in the pediatrics department, diagnosis of Cushing’s disease, pharmacological steroid use at the time of CRH testing, and measurement of serum cortisol with a kit from Siemens Healthcare K.K. (Tokyo, Japan (19)) utilizing a polyclonal antibody. All available CRH stimulation test data were analyzed, although some time points were missing. Patients were classified into the HC (+) or HC (–) group based on the requirement for HC replacement treatment, as recorded in the registry. For patients who underwent surgery or radiotherapy involving the pituitary gland or hypothalamus, only data obtained prior to the intervention or at least 1 month postoperatively were included. Based on the sensitivity and specificity estimates derived from the cortisol cutoff value yielding the highest AUC in the discovery cohort, the sample size was calculated to ensure 80% power with a one-sided significance level of 15%. As a result, an estimated 53 and 43 patients were required to validate the sensitivity and specificity, respectively.

### CRH stimulation test protocol

In the discovery cohort, the CRH stimulation test was started at 09:00 after fasting overnight (12 hours) and at least 30 minutes of bed rest. In the supine position, 100 µg human CRH (corticorelin) was administered intravenously, and blood samples were collected before injection and at 30, 60, 90, and 120 minutes after injection for plasma ACTH and serum cortisol measurements. The blood samples were kept cold and sent promptly to the laboratory. For patients receiving HC, the drug was stopped at least 12 hours prior to the test. The 12-hour discontinuation period was based on data from several published reports, taking into account hydrocortisone’s plasma half-life of 80 minutes and biological half-life of 8 to 12 hours, with the aim of minimizing the risk of adrenal crisis (20–23). In the validation cohort, testing was conducted following each institution’s protocol.

### ACTH and cortisol assays

In the discovery cohort, plasma ACTH and serum cortisol concentrations were measured by electrochemiluminescence immunoassay (Elecsys ACTH and Cortisol II, Roche Diagnostics K.K., Tokyo, Japan (24, 25)). In the validation cohort, these concentrations were measured using monoclonal antibody-based commercial kits at each institution. The intra- and inter-assay variability, as well as the cross-reactivity for cortisone, of the measurement kits used in this study are described in **Supplementary Table S1**.

### Statistical analysis

Continuous data are expressed as medians with the interquartile range (IQR) and categorical data as numbers. Continuous data were assessed for normality using the Shapiro–Wilk test. Normally distributed data were analyzed using the 2–sample t–test. Non-normally distributed data were analyzed using the Mann–Whitney test and categorical data using the chi-square test. Statistical analyses were performed using IBM SPSS Statistics 28 (IBM, Armonk, NY, USA). Significance was defined as a p value < 0.05. Scatter plots were created in GraphPad Prism, version 9 (GraphPad Software, Inc., La Jolla, CA, USA). To determine cutoff plasma ACTH and serum cortisol levels, receiver operating characteristic (ROC) analysis was performed using JMP 18 (SAS Institute Inc., Cary, NC, USA), with the HC (–) group treated as positive. Diagnostic accuracy was defined as (true positives + true negatives)/total number × 100 (26). Sensitivity, specificity, and diagnostic accuracy in the validation cohort were calculated for each cutoff value obtained in the discovery cohort.

## Results

### Patient characteristics in the discovery cohort

Of the 227 patients included in the discovery cohort, 91 required HC supplementation for ACTH deficiency [HC (+) group], while 136 did not [HC (–) group] (**Table 1**). All 91 patients in the HC (+) group were initially assessed as requiring HC at baseline, and no additional patients initiated HC therapy during follow-up. Conversely, 13 patients in the HC (–) group were initially assessed as requiring HC but later discontinued treatment based on follow-up clinical information. Compared with the HC (–) group, the HC (+) group had a significantly higher proportion of males (p < 0.001), older age (p < 0.001), and longer follow-up period (p = 0.002). The underlying etiologies of ACTH deficiency of patients in the HC (+) group and the background characteristics of patients in the HC (–) group are shown in **Tables 2** and **3**, respectively.

**Table 1:** Clinical characteristics of patients in the discovery cohort.

Variables	HC (–)	HC (+)	p value
	(n = 136)	(n = 91)	
Sex, male/female, n	52/84	57/34	**< 0.001**
Age, years	42 (28–57)	68 (54–75)	**< 0.001**
Follow-up period, days	418 (84–1548)	957 (366–1704)	**0.002**
Body mass index, kg/m^2^	22.4 (19.7–26.5)	22.6 (19.4–26.5)	0.788

Values are numbers or medians (interquartile range). p values < 0.05 are shown in bold. HC (–), patients not requiring hydrocortisone treatment; HC (+), patients requiring hydrocortisone treatment.

**Table 2:** Underlying etiologies of ACTH deficiency in patients treated with hydrocortisone in the discovery cohort.

Variables	Number of patients
Sellar or suprasellar diseases^1)^	24
ICI-related pituitary dysfunction	51
Idiopathic pituitary dysfunction	13
IgG4-related disease	3

ICI, immune checkpoint inhibitor. ^1)^Acromegaly (n = 6), Rathke’s cleft cyst (n = 5), craniopharyngioma (n = 3), germinoma (n = 3), pituitary stalk interruption (n = 2), after surgery for Cushing’s disease (n = 2), non-functioning pituitary tumor (n = 1), hemangioblastoma (n = 1), Sheehan’s syndrome (n = 1).

**Table 3:** Background characteristics of patients who did not require hydrocortisone therapy in the discovery cohort.

Variables	Number of patients
Symptoms or laboratory findings suggestive of hypopituitarism^1)^	61
Pituitary dysfunction without ACTH deficiency^2)^	10
Sellar or suprasellar lesions^3)^	54
Intracranial diseases (other than sellar lesions)^4)^	6
IgG4-related disease	1
Others^5)^	4

ICI, immune checkpoint inhibitor; ACTH, adrenocorticotropic hormone. ^1)^4 Patients were treated with ICI. ^2)^Central hypogonadism (n = 5), growth hormone deficiency (n = 2), central diabetes insipidus (n = 1), central hypothyroidism (n = 1), hyperprolactinemia (n = 1). ^3)^Acromegaly (n = 20), non-functioning pituitary tumor (n = 9), Rathke’s cleft cyst (n = 9), prolactinoma (n = 4), craniopharyngioma (n = 2), pituitary gland enlargement (n = 2), TSH-secreting pituitary tumor (n = 2), arachnoid cyst (n = 1), cavernous hemangioma (n = 1), empty sella (n = 1), pituitary apoplexy (n = 1), pituitary atrophy (n = 1), pituitary metastasis of lung adenocarcinoma (n = 1). ^4)^Germ cell tumor in the basal ganglia (n = 1), germ cell tumor in the pineal gland (n = 1), cerebral ventricle tumor (n = 1), infratentorial ependymoma (n = 1), medulloblastoma in the cerebellum (n = 1), 4H syndrome (n = 1). ^5)^Langerhans cell histiocytosis (n = 2), Erdheim–Chester disease (n =1), pachydermoperiostosis (n = 1).

### Cortisol and ACTH responses to CRH stimulation in the HC (–) and HC (+) groups

**Figure 2:**
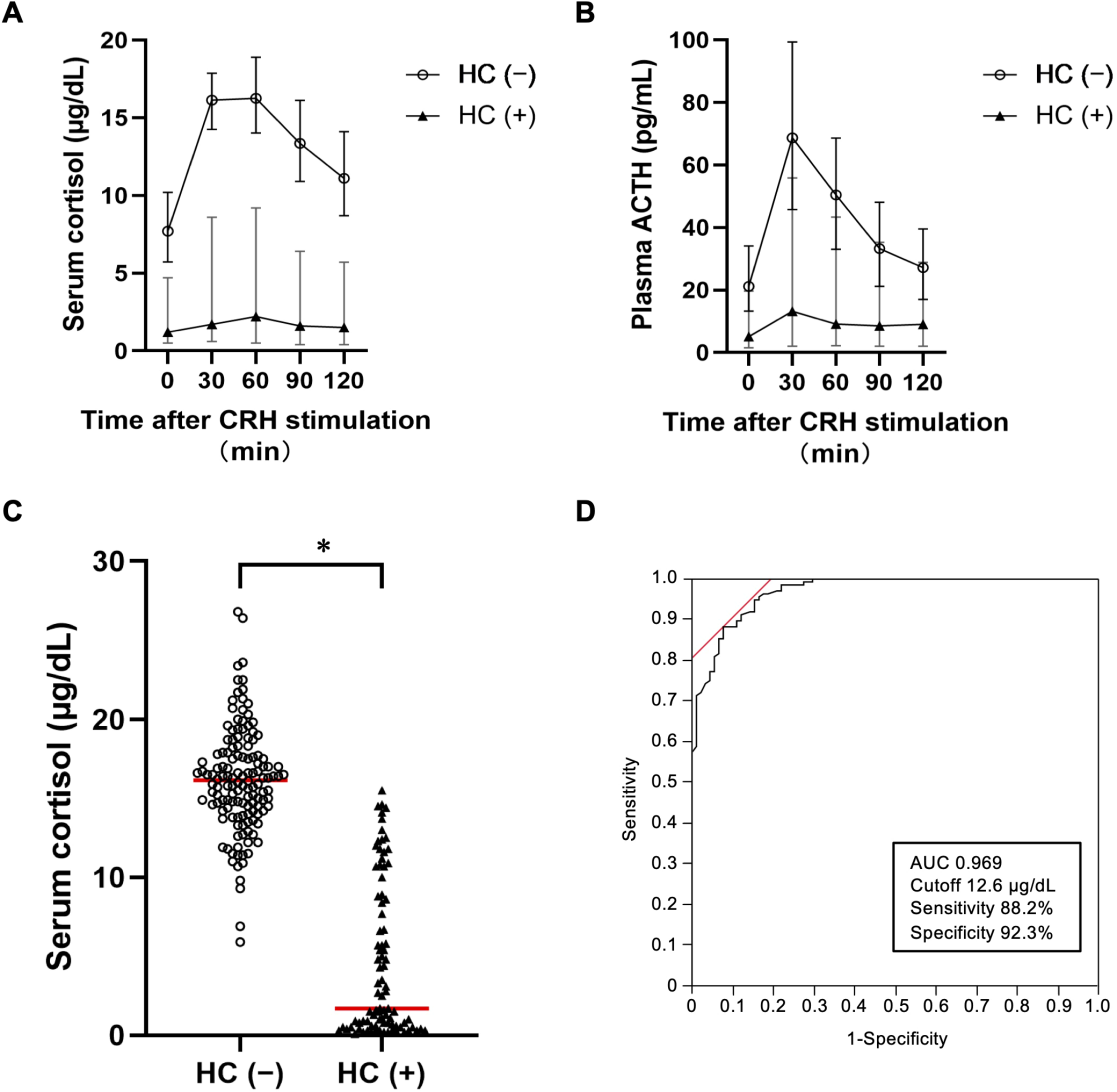
Time-course changes in serum cortisol and plasma ACTH levels after corticotropin-releasing hormone (CRH) stimulation in the discovery cohort. **(A, B)** Time-course changes in serum cortisol **(A)** and plasma ACTH **(B)** levels following CRH stimulation in the HC (–) and HC (+) groups. Open circles and closed triangles represent the median serum cortisol level at each time point in the HC (–) and HC (+) group, respectively. Whiskers extend from the 25th to 75th percentiles. **(C)** Serum cortisol levels at 30 minutes post-CRH stimulation in the HC (–) group (open circle) and HC (+) group (closed triangle): 16.2 [14.3–17.9] vs. 1.7 [0.6–8.6] µg/dL (p < 0.001). **(D)** Receiver operating characteristic analysis of the optimal serum cortisol level at 30 minutes post-CRH stimulation predicting the need for HC treatment. The 95% confidence interval for the AUC was 0.950–0.987. To convert the units of serum cortisol levels from µg/dL to nmol/L, multiply by 27.6. *p < 0.001. HC (–), patients not requiring hydrocortisone treatment; HC (+), patients requiring hydrocortisone treatment; CRH, corticotropin-releasing hormone; ACTH, adrenocorticotropic hormone; AUC, area under the curve.

The time-course changes in serum cortisol levels following CRH stimulation in the HC (–) and HC (+) groups are shown in **Figure 2A**. Cortisol levels peaked at 30 and 60 minutes post-stimulation (16.2 and 16.3 µg/dL [446.9 and 449.7 nmol/L], respectively) in the HC (–) group. The values were significantly higher in the HC (–) group than in the HC (+) group at all measured time points (**Figure 2C**, **Supplementary Figure S1A–E**).

### Diagnostic performance of the cortisol level at 30 minutes post-CRH stimulation

Based on the ROC analysis, the cortisol level at 30 minutes post-CRH stimulation demonstrated the highest discriminatory performance, with an optimal cutoff value of 12.6 µg/dL (347.6 nmol/L) (AUC: 0.969 [95% confidence interval, 0.950−0.987]; sensitivity: 88.2%; specificity: 92.3%; accuracy: 89.9%) (**Figure 2D**). Cortisol levels at the other time points resulted in lower AUCs (**Supplementary Figure S1F–J**). The conventionally used cutoff of 18 µg/dL also had weaker diagnostic performance (sensitivity: 36.8%; specificity: 98.9%; accuracy: 61.7%) compared with our cutoff of 12.6 µg/dL.

### Diagnostic performance of the ACTH level post-CRH stimulation

The time-course changes in plasma ACTH levels following CRH stimulation in the HC (–) and HC (+) groups are shown in **Figure 2B**, respectively. The ACTH level peaked at 30 minutes post-stimulation in the HC (–) group and was significantly higher in the HC (–) than HC (+) group at all measured time points (**Supplementary Figure S2A–F**). The ACTH level at 30 minutes post-CRH stimulation had the highest AUC (0.809) among all time points evaluated (**Supplementary Figure S2H**). However, the diagnostic accuracy of ACTH was lower than that of cortisol at all time points (**Supplementary Figure S2G–L**).

### Patient characteristics in the validation cohort

Among the 52 patients in the validation cohort, 25 were in the HC (+) group and 27 were in the HC (–) group (**Supplementary Table S2**). There was no significant difference in sex, age, or body mass index between the two groups. The underlying etiologies of the patients in both groups are shown in **Supplementary Table S3**. Serum cortisol levels were measured using kits provided by Roche Diagnostics K.K. (n = 36), Tosoh Corporation (Tokyo, Japan (27, 28)) (n = 12), and FUJIFILM Wako Pure Chemical Corporation (Osaka, Japan (29)) (n = 4).

### Confirmation of the diagnostic performance of the 30-minute cortisol level in the validation cohort

**Figure 3:**
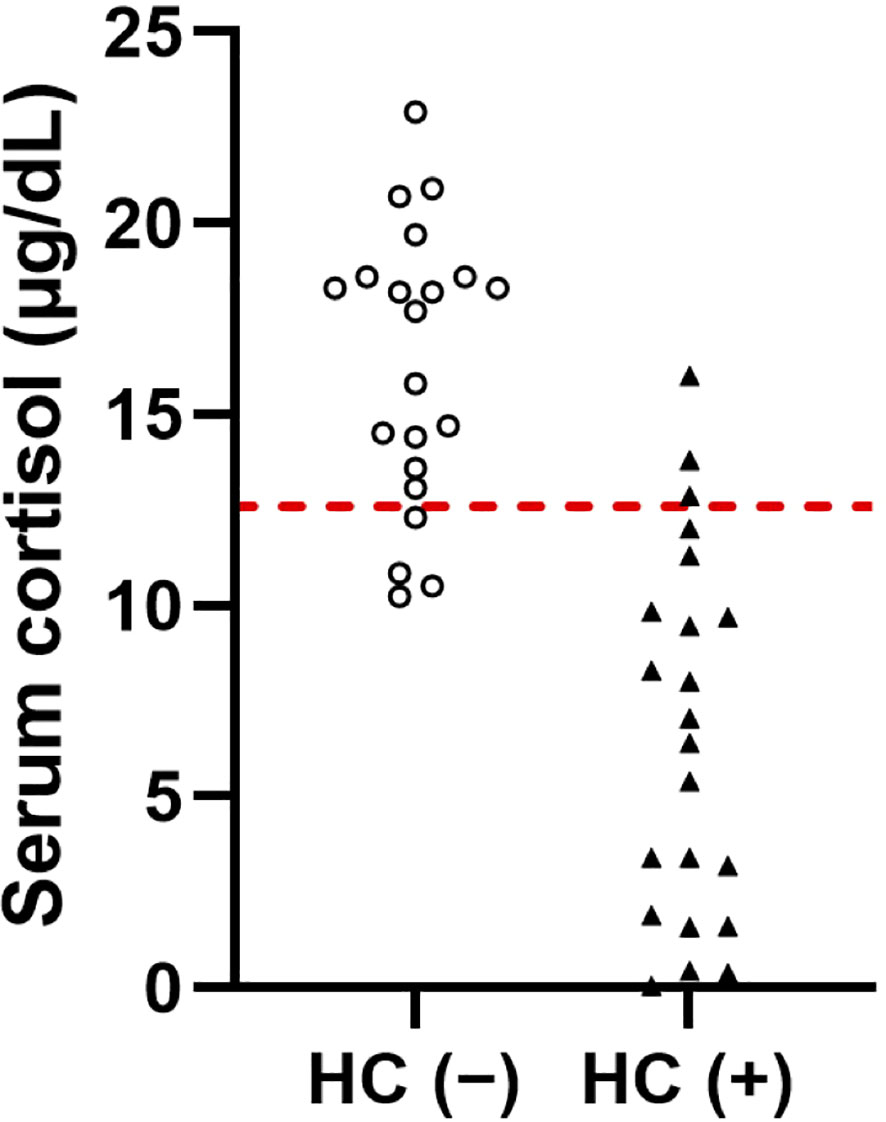
Serum cortisol levels at 30 minutes post-CRH stimulation in the validation cohort. The red dashed line indicates a serum cortisol level of 12.6 µg/dL. To convert the units of serum cortisol levels from µg/dL to nmol/L, multiply by 27.6. HC (–), patients not requiring hydrocortisone treatment; HC (+), patients requiring hydrocortisone treatment.

When the cutoff values derived from the discovery cohort were applied to each corresponding time point in the validation cohort, the cortisol level at 30 minutes (≥ 12.6 µg/dL) again demonstrated the highest diagnostic performance compared with the other time points (sensitivity: 81.0%; specificity: 86.4%; accuracy: 83.7%; **Figure 3**, **Supplementary Table S4**). The ACTH cutoff level at each time point again performed worse than the cortisol cutoff level (**Supplementary Table S4**).

## Discussion

In this retrospective single-center study with validation using Japanese nationwide data, we found that a 30-minute post-CRH cortisol level <12.6 µg/dL provided high diagnostic accuracy for identifying patients who require HC replacement. This proposed cutoff may guide clinical evaluation of ACTH deficiency.

Central adrenal insufficiency is commonly diagnosed based on the basal cortisol level and cortisol response to ITT or CRH stimulation testing (1–7); in particular, a serum cortisol level < 18 µg/dL has traditionally been used as the cutoff indicating impaired response (3, 5–7). However, this cutoff was established based on an ITT study reporting a peak cortisol range of 18–30 µg/dL in healthy individuals or patients without endocrine disorders (9). Although ITT has long been regarded as the gold standard for evaluating HPA axis function, it requires careful monitoring to ensure patient safety and is less feasible in routine clinical practice (1, 3–7). Furthermore, in this study, 63.2% (86/136) of patients in the HC (–) group exhibited a peak cortisol level < 18 µg/dL post-CRH stimulation yet did not require HC replacement therapy during follow-up, suggesting limited diagnostic utility of the conventional cutoff of 18 µg/dL.

When establishing cortisol cutoffs, it is crucial to consider changes in the assay methods. Since 2016 in many countries including Japan, specific monoclonal antibody-based assays have started to replace previously used polyclonal antibody-based immunoassays for cortisol measurements (10–12). The new assays yield approximately 20% lower cortisol values compared with the previous methods (13, 14). For example, the cortisol level of 18 µg/dL detected by the previous Roche Elecsys^®^ Cortisol I immunoassay corresponds to 14.5 µg/dL (400 nmol/L) by LC-MS/MS, 14.6 µg/dL (402.8 nmol/L) by the Roche Elecsys^®^ Cortisol II immunoassay (used in our discovery cohort), and 14.8 µg/dL (408.3 nmol/L) by Beckman Access^®^ cortisol assay (Beckman Coulter, Brea, CA, USA) (13). A recent study involving healthy individuals (n = 100) proposed reference ranges for the serum cortisol level at 30 minutes after 250 µg ACTH stimulation based on 2.5th and 97.5th percentiles: 435–710 nmol/L (15.8–25.7 µg/dL; Elecsys^®^ Cortisol II) and 426–709 nmol/L (15.4–25.7 µg/dL; LC-MS/MS), and adjusted 2.5th percentile values as lower limits: 426 nmol/L (15.4 µg/dL) (Elecsys^®^ Cortisol II) and 411 nmol/L (14.9 µg/dL) (LC-MS/MS) (14). However, it remains unclear whether the cortisol cutoff values from ACTH stimulation tests can be applied to the CRH stimulation test, or whether the lower cortisol level limit derived from healthy individuals can serve as a diagnostic cutoff for ACTH deficiency.

Several reports have examined cutoff values for CRH stimulation testing to diagnose central adrenal insufficiency. Schmidt et al. provided a post-stimulation cortisol cutoff of 377 nmol/L (13.7 µg/dL) (76% sensitivity, 96% specificity) to diagnose central adrenal insufficiency, defined as a peak cortisol level < 500 nmol/L in the ITT, in 54 patients with hypothalamic–pituitary–adrenal axis disorders (30). In another retrospective cohort study by Mitsui et al. involving 215 Japanese patients, ROC analysis identified a peak post-CRH stimulation cortisol cutoff ≥ 17.5 µg/dL as indicating no need for HC replacement, whereas < 10.0 µg/dL (275.9 nmol/L) indicated a need for replacement therapy (18). However, ACTH deficiency was diagnosed based on a peak cortisol level < 500 nmol/L in both studies. Furthermore, serum cortisol levels were measured by conventional polyclonal antibody-based assays in the Schmidt et al. study (30), while both conventional polyclonal and newer monoclonal antibody-based assays were employed in the Mitsui et al. study (18). To the best of our knowledge, this is the first study to determine a cortisol cutoff level post-CRH stimulation for diagnosing ACTH deficiency based exclusively on monoclonal antibody-based assay data.

Basal cortisol levels < 3–5 µg/dL (82.8–137.9 nmol/L) are reportedly useful for diagnosing adrenal insufficiency (1, 3–5, 7, 30). Although a basal cortisol cutoff level (4.3 µg/dL [118.6 nmol/L]) comparable with previously reported values was identified in this study, its specificity (72.5%) was lower than that of the 30-minute post-CRH stimulation cortisol level. The plasma ACTH level has limited value for diagnosing central adrenal insufficiency (1, 5–7), one reason being that plasma ACTH is unstable and highly influenced by collection procedures and storage conditions (6, 31, 32). Moreover, central adrenal insufficiency includes pituitary and hypothalamic types, which exhibit different ACTH responses to CRH stimulation (1, 3, 33). In this study, the diagnostic value of ACTH was lower than that of cortisol.

In the discovery cohort of this study, there were significant differences in the duration of the follow-up periods between the HC (+) and (–) groups. The difference in follow-up duration may be explained by the fact that the HC (+) group required treatment, whereas the HC (–) group did not, and therefore, the HC (–) group completed follow-up earlier. Although no patient initially judged not to require HC later required it at the final follow-up, this difference suggests that a shorter follow-up period could have limited the opportunity to reassess and initiate HC treatment.

This study has several limitations. First, the proposed cortisol cutoff was based on a relatively small number of patients (n = 227) in the discovery cohort. Second, the decision regarding HC replacement was based on the clinical judgment of the attending endocrinologists. Although it was determined not only by the results of the CRH test, clinical symptoms, and general laboratory findings at baseline, but also by the clinical information obtained during the follow-up period (with a median duration of more than one year in both groups), the conventional cortisol cutoff (18 µg/dL) might have influenced their decisions. Third, ITT was not performed in most patients in the discovery cohort due to safety concerns. It will be important to compare ITT responses with those on the CRH test in future research. Fourth, there were differences in sex and age between the HC (+) and HC (–) groups in the discovery cohort. Future studies are warranted to adjust for these baseline characteristics. Fifth, although all cortisol levels in the validation cohort were measured by current cortisol assays utilizing monoclonal antibodies, the assay kits differed among institutions. It is desirable to establish assay-specific cutoffs or adjust cutoff thresholds to account for inter-assay variability in the future. The strength of this study is that the cortisol cutoff was validated by data from a Japanese nationwide registry.

In conclusion, a 30-minute post-CRH cortisol level of 12.6 µg/dL may be useful for diagnosing ACTH deficiency.

## Data Availability

The original contributions presented in the study are included in the article/**Supplementary Material**. Further inquiries can be directed to the corresponding author/s.

## Supplementary material

Supplementary Figure 1 Receiver operating characteristic analysis of serum cortisol levels post-CRH stimulation at different time points. Serum cortisol levels at 0 **(A)**, 60 **(B)**, 90 **(C)**, and 120 **(D)** minutes and the peak level **(E)** post-CRH stimulation. Receiver operating characteristic analyses at 0 **(F)**, 60 **(G)**, 90 **(H)**, and 120 **(I)** minutes and the peak level **(J)** post-CRH stimulation. Cortisol levels were significantly higher in the HC (–) than HC (+) group: 7.7 [5.7–10.2] vs. 1.2 [0.5–4.7] µg/dL at 0 minutes (p < 0.001), 16.3 [14.0–18.9] vs. 2.2 [0.5–9.2] µg/dL at 60 minutes(p < 0.001), 13.4 [10.9–16.1] vs. 1.6 [0.4–6.4] µg/dL at 90 minutes (p < 0.001), 11.1 [8.7–14.1] vs. 1.5 [0.4–5.7] µg/dL at 120 minutes (p < 0.001), and 17.3 [15.1–19.5] vs. 2.2 [0.6–9.2] µg/dL at the peak time point (p < 0.001). The 95% confidence interval for the AUC at each time point was as follows: 0.867–0.950 at 0 minutes, 0.912–0.977 at 60 minutes, 0.879–0.965 at 90 minutes, 0.855–0.952 at 120 minutes, and 0.924–0.984 at the peak time point. To convert the units of serum cortisol levels from µg/dL to nmol/L, multiply by 27.6. * p < 0.001. HC (–), patients not requiring hydrocortisone treatment; HC (+), patients requiring hydrocortisone treatment; CRH, corticotropin-releasing hormone.

Supplementary Figure 2 Receiver operating characteristic analysis of plasma ACTH levels post-CRH stimulation at different time points. Plasma ACTH levels at 0 **(A)**, 30 **(B)**, 60 **(C)**, 90 **(D)**, and 120 **(E)** minutes and the peak level **(F)** after CRH stimulation. Receiver operating characteristic analysis at 0 **(G)**, 30 **(H)**, 60 **(I)**, 90 **(J)**, and 120 **(K)** minutes and the peak level **(L)** post-CRH stimulation. ACTH levels were significantly higher in the HC (–) than HC (+) group: 21.2 [13.3–34.1] vs. 5.1 [1.5–19.7] pg/mL at 0 minutes (p < 0.001), 68.7 [45.9–99.4] vs. 13.2 [2.0–55.9] pg/mL at 30 minutes (p < 0.001), 50.5 [33.0–68.6] vs. 9.1 [2.2–43.4] pg/mL at 60 minutes (p < 0.001), 33.3 [21.2–48.1] vs. 8.5 [2.0–35.3] pg/mL at 90 minutes (p < 0.001), 27.2 [17.0–39.5] vs. 9.0 [2.0–28.9] pg/mL at 120 minutes (p < 0.001), and 69.9 [50.9–99.3] vs. 14.2 [2.7–63.4] pg/mL at the peak time point (p < 0.001). The 95% confidence interval for the AUC at each time point was as follows: 0.728–0.854 at 0 minutes, 0.746–0.873 at 30 minutes, 0.702–0.844 at 60 minutes, 0.671–0.818 at 90 minutes, 0.655–0.803 at 120 minutes, 0.726–0.861 at the peak time point. * p < 0.001. ACTH, adrenocorticotropic hormone; HC (–), patients not requiring hydrocortisone treatment; HC (+), patients requiring hydrocortisone treatment; CRH, corticotropin-releasing hormone.
