# Supplementary figures and images for: Defining a serum cortisol cutoff level post-CRH stimulation for diagnosing ACTH deficiency: A retrospective study validated by a nationwide registry
Source: Front Endocrinol (Lausanne). 2026 Feb 2;17:1741709. doi: 10.3389/fendo.2026.1741709 (PMC12907428; doi:10.3389/fendo.2026.1741709)

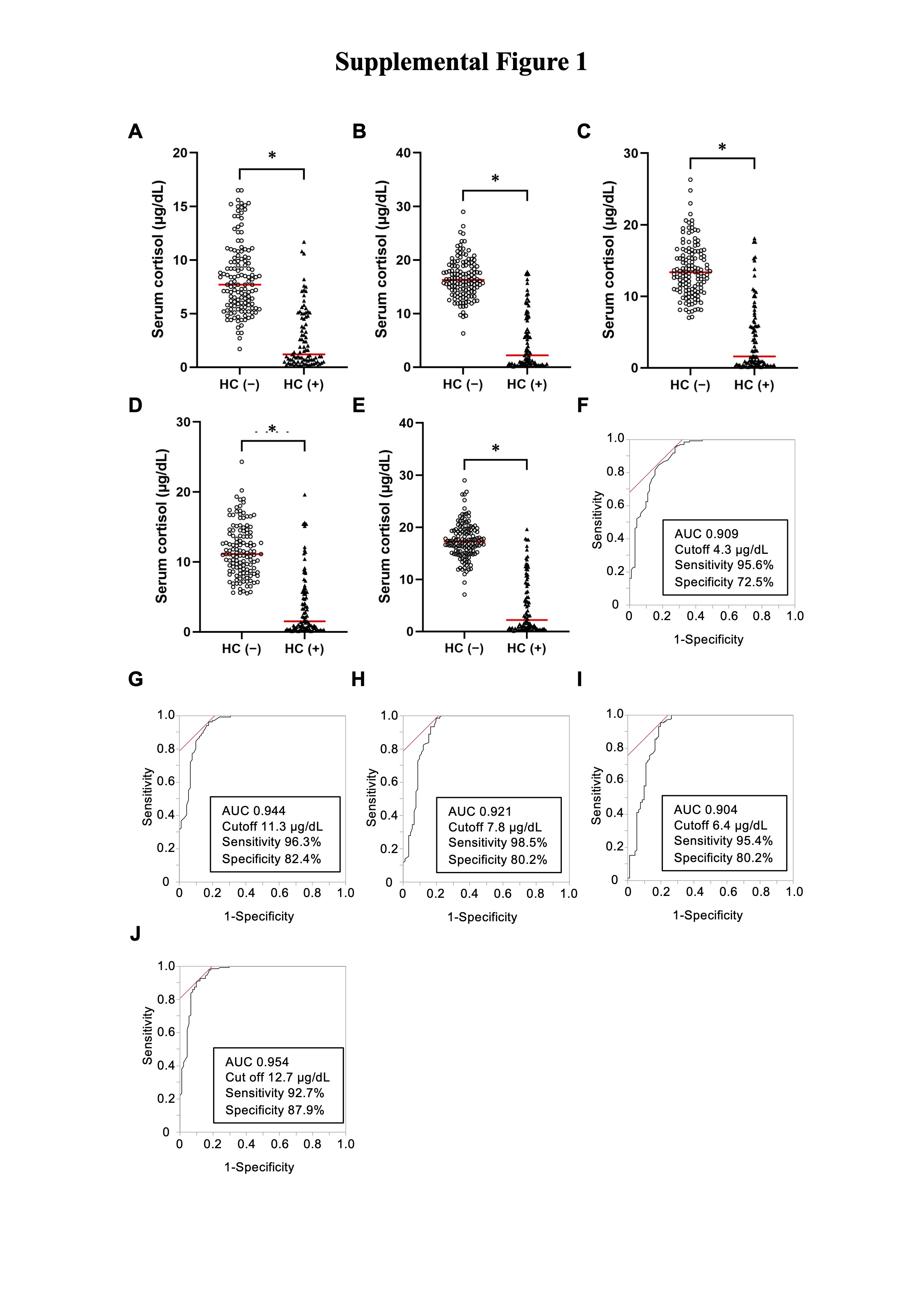

Supplement: Supplementary Figure 1 — Receiver operating characteristic analysis of serum cortisol levels post-CRH stimulation at different time points. Serum cortisol levels at 0 (A), 60 (B), 90 (C), and 120 (D) minutes and the peak level (E) post-CRH stimulation. Receiver operating characteristic analyses at 0 (F), 60 (G), 90 (H), and 120 (I) minutes and the peak level (J) post-CRH stimulation. Cortisol levels were significantly higher in the HC (–) than HC (+) group: 7.7 [5.7–10.2] vs. 1.2 [0.5–4.7] µg/dL at 0 minutes (p < 0.001), 16.3 [14.0–18.9] vs. 2.2 [0.5–9.2] µg/dL at 60 minutes(p < 0.001), 13.4 [10.9–16.1] vs. 1.6 [0.4–6.4] µg/dL at 90 minutes (p < 0.001), 11.1 [8.7–14.1] vs. 1.5 [0.4–5.7] µg/dL at 120 minutes (p < 0.001), and 17.3 [15.1–19.5] vs. 2.2 [0.6–9.2] µg/dL at the peak time point (p < 0.001). The 95% confidence interval for the AUC at each time point was as follows: 0.867–0.950 at 0 minutes, 0.912–0.977 at 60 minutes, 0.879–0.965 at 90 minutes, 0.855–0.952 at 120 minutes, and 0.924–0.984 at the peak time point. To convert the units of serum cortisol levels from µg/dL to nmol/L, multiply by 27.6. * p < 0.001. HC (–), patients not requiring hydrocortisone treatment; HC (+), patients requiring hydrocortisone treatment; CRH, corticotropin-releasing hormone. [file Image1.jpeg]

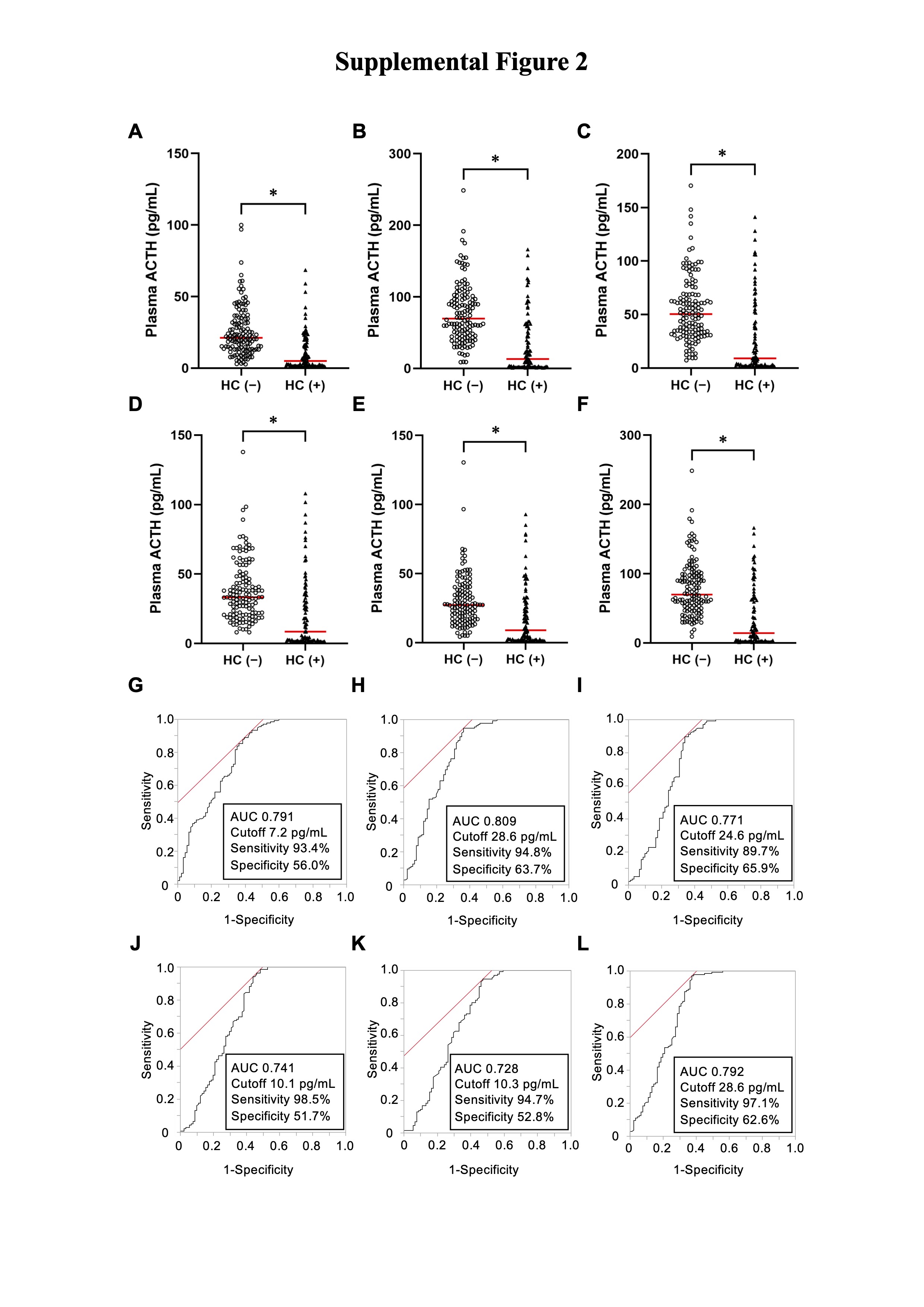

Supplement: Supplementary Figure 2 — Receiver operating characteristic analysis of plasma ACTH levels post-CRH stimulation at different time points. Plasma ACTH levels at 0 (A), 30 (B), 60 (C), 90 (D), and 120 (E) minutes and the peak level (F) after CRH stimulation. Receiver operating characteristic analysis at 0 (G), 30 (H), 60 (I), 90 (J), and 120 (K) minutes and the peak level (L) post-CRH stimulation. ACTH levels were significantly higher in the HC (–) than HC (+) group: 21.2 [13.3–34.1] vs. 5.1 [1.5–19.7] pg/mL at 0 minutes (p < 0.001), 68.7 [45.9–99.4] vs. 13.2 [2.0–55.9] pg/mL at 30 minutes (p < 0.001), 50.5 [33.0–68.6] vs. 9.1 [2.2–43.4] pg/mL at 60 minutes (p < 0.001), 33.3 [21.2–48.1] vs. 8.5 [2.0–35.3] pg/mL at 90 minutes (p < 0.001), 27.2 [17.0–39.5] vs. 9.0 [2.0–28.9] pg/mL at 120 minutes (p < 0.001), and 69.9 [50.9–99.3] vs. 14.2 [2.7–63.4] pg/mL at the peak time point (p < 0.001). The 95% confidence interval for the AUC at each time point was as follows: 0.728–0.854 at 0 minutes, 0.746–0.873 at 30 minutes, 0.702–0.844 at 60 minutes, 0.671–0.818 at 90 minutes, 0.655–0.803 at 120 minutes, 0.726–0.861 at the peak time point. * p < 0.001. ACTH, adrenocorticotropic hormone; HC (–), patients not requiring hydrocortisone treatment; HC (+), patients requiring hydrocortisone treatment; CRH, corticotropin-releasing hormone. [file Image2.jpeg]
