# Supplementary material for: Defining a serum cortisol cutoff level post-CRH stimulation for diagnosing ACTH deficiency: A retrospective study validated by a nationwide registry
Source: Front Endocrinol (Lausanne). 2026 Feb 2;17:1741709. doi: 10.3389/fendo.2026.1741709 (PMC12907428; doi:10.3389/fendo.2026.1741709)
Supplement: Supplementary file 3 [file Table1.doc]

**Supplemental Table 1. Characteristics of measurement kits used in this study.**

| **Name of measurement kit** | **Intra-assay CV** | **Inter-assay CV** | **Cross-reactivity for cortisone** | **Reference** |
| --- | --- | --- | --- | --- |
| Elecsys ACTH (Roche Diagnostics) | ≦10% | ≦25% | Not applicable | (1) |
| Elecsys Cortisol II (Roche Diagnostics) | ≦15% | ≦20% | 4.68% for 10 µg/mL | (2) |
|  | 1.0–1.7% | 2.2–2.8% | Not available | (3) |
| AIA Cortisol (Tosoh Bioscience) | ≦15% | ≦20% | 1.95% for 10 µg/mL | (4) |
| AIA-pack CL Cortisol (Tosoh Bioscience) | ≦15% | ≦20% | 2.57% for 10 µg/mL | (5) |
| AcuraSeed Cortisol (FUJIFILM Wako Pure Chemical Corporation) | ≦10% | ≦15% | Not available | (6) |

**Abbreviations**: CV, coefficient of variation.

**References**

**Supplemental Table 2. Clinical characteristics of patients in the validation cohort**

|  | **HC (–)** | **HC (+)** | **p value** |
| --- | --- | --- | --- |
|  | **(n = 27)** | **(n = 25)** |  |
| Sex, male/female, n | 14/13 | 16/19 | 0.376 |
| Age, years | 51 (32–67) | 58 (41–66) | 0.838 |
| Body mass index, kg/m^2^ | 22.8 (21.5–26.4) | 22.8 (21.3–27.3) | 0.873 |

Values are numbers or medians (interquartile range). **Abbreviations:** HC (–), patients not requiring hydrocortisone treatment; HC (+), patients requiring hydrocortisone treatment.

**Supplemental Table 3. Underlying diseases in patients in the validation cohort**

|  | **HC (–)** | **HC (+)** |
| --- | --- | --- |
|  | **(n = 27)** | **(n = 25)** |
| Sellar or suprasellar diseases | 23^1)^ | 23^2)^ |
| ICI-related pituitary dysfunction | 0 | 1 |
| Idiopathic pituitary dysfunction | 3 | 0 |
| IgG4-related disease | 1 | 1 |

**Abbreviations**: HC (–), patients not requiring hydrocortisone treatment; HC (+), patients requiring hydrocortisone treatment; ICI, immune checkpoint inhibitor. ^1)^Acromegaly (n = 11), non-functioning pituitary tumor (n = 8), TSH-secreting pituitary tumor (n = 3), Rathke’s cleft cyst (n = 1). ^2)^Craniopharyngioma (n = 7), non-functioning pituitary tumor (n = 6), acromegaly (n = 4), Rathke’s cleft cyst (n = 3), empty sella (n = 1), Sheehan’s syndrome (n = 1), after whole brain radiation therapy for cerebellar tumor (n = 1).

**Supplemental Table 4. Accuracy of different cutoff values for diagnosing ACTH deficiency in the validation cohort**

| Time | 0 min | 30 min | 60 min | 90 min | peak |
| --- | --- | --- | --- | --- | --- |
| Cutoff plasma ACTH (pg/mL) | 8.6 | 28.6 | 24.6 | 14.1 | 28.6 |
| Sensitivity (%) | 96.3 | 96.3 | 92.6 | 94.7 | 96.3 |
| Specificity (%) | 32.0 | 24.0 | 24.0 | 18.2 | 24.0 |
| Accuracy (%) | 65.4 | 61.5 | 59.6 | 53.7 | 61.5 |
| Cutoff serum cortisol (µg/dL) | 4.3 | 12.6 | 11.3 | 7.8 | 12.7 |
| Sensitivity (%) | 92.6 | 81.0 | 85.2 | 96.3 | 85.2 |
| Specificity (%) | 60.0 | 86.4 | 68.0 | 64.0 | 72.0 |
| Accuracy (%) | 76.9 | 83.7 | 76.9 | 80.8 | 78.8 |

To convert the units of serum cortisol levels from µg/dL to nmol/L, multiply by 27.6. **Abbreviations**: ACTH, adrenocorticotropic hormone.
